# Atypical Gastrointestinal Kaposi Sarcoma With Circumferential Anorectal Involvement in a Young Man With Controlled HIV Infection: A Case Report

**DOI:** 10.7759/cureus.108899

**Published:** 2026-05-15

**Authors:** Maria H García Ramírez, Laura B Alegria Lopez, Isac I Ramírez-Preciado, Luis A Villalobos Calderon, Juan G Garcia Gonzalez

**Affiliations:** 1 General Surgery, Centro Médico Nacional de Occidente, Instituto Mexicano de Seguro Social (IMSS), Guadalajara, MEX; 2 Colorectal Surgery, Centro Médico Nacional de Occidente, Instituto Mexicano de Seguro Social (IMSS), Guadalajara, MEX; 3 General Surgery, Instituto de Seguridad y Servicios Sociales de los Trabajadores del Estado, Hospital de Especialidades, Morelia, MEX; 4 Oncologic Surgery, Private Practice, Morelia, MEX

**Keywords:** acute rectal bleeding, anorectal disease, gastrointestinal kaposi sarcoma, kaposi sarcoma management, colonoscopy

## Abstract

Gastrointestinal Kaposi sarcoma is an uncommon but clinically relevant manifestation of human herpesvirus 8-associated disease in patients with human immunodeficiency virus (HIV) infection. Its diagnosis may be delayed because symptoms are often nonspecific and may overlap with other anorectal disorders, particularly in patients with condylomatous disease, fissures, proctitis, or anal malignancy. We report the case of a 25-year-old male with HIV infection on antiretroviral therapy and an undetectable viral load who presented with one month of anal pain and rectal bleeding. Initial colonoscopy demonstrated multiple irregular ulcerated lesions in the distal rectum, and biopsy confirmed Kaposi sarcoma associated with human herpesvirus 8. Despite referral to Medical Oncology and initiation of chemotherapy, the patient had persistent rectal bleeding, mucus discharge, anal condyloma, and fissure. Repeat proctologic evaluation and colonoscopy revealed elevated, irregular, ulcerated circumferential lesions extending from the anal canal to 10 cm proximally, with increased submucosal vascular pattern. Persistent gastrointestinal Kaposi sarcoma with suspicious anorectal tumoral involvement was identified, and the patient was referred for multidisciplinary oncologic management. This case highlights the diagnostic challenge of anorectal Kaposi sarcoma in patients with HIV infection, especially when symptoms overlap with benign anorectal disease or synchronous malignancy. Early endoscopic evaluation, biopsy, and immunohistochemistry are essential for timely diagnosis and appropriate multidisciplinary treatment.

## Introduction

Kaposi sarcoma is a low-grade angioproliferative neoplasm associated with human herpesvirus 8 infection and represents one of the characteristic malignancies linked to human immunodeficiency virus (HIV)-related immunosuppression [1]. Four clinical variants have been described: classic, endemic, iatrogenic or immunosuppression-related, and AIDS-related Kaposi sarcoma [1,2]. Although the widespread use of antiretroviral therapy has reduced the incidence of AIDS-related Kaposi sarcoma, visceral involvement remains clinically relevant and may be difficult to recognize when cutaneous lesions are absent or when symptoms are dominated by nonspecific gastrointestinal complaints [2,3]. Since the introduction of highly active antiretroviral therapy, the incidence of HIV-associated Kaposi sarcoma has significantly declined, particularly among patients with adequate treatment adherence and immune reconstitution. Nevertheless, Kaposi sarcoma remains one of the most common HIV-associated malignancies and may still occur despite virologic suppression and preserved CD4 counts [2].

Gastrointestinal involvement may affect any segment from the oropharynx to the rectum. Clinical manifestations range from asymptomatic disease to diarrhea, abdominal pain, nausea, vomiting, weight loss, iron-deficiency anemia, and overt gastrointestinal bleeding [2-4]. Less commonly, gastrointestinal Kaposi sarcoma may present with severe complications such as intestinal obstruction, intussusception, massive hemorrhage, or perforation [3,5,6]. The endoscopic appearance is also variable and may include erythematous or violaceous maculopapular lesions, nodules, polypoid lesions, ulcerations, and mass-like lesions with friability or contact bleeding [2,4,7].

The diagnosis of gastrointestinal Kaposi sarcoma requires endoscopic evaluation with biopsy and immunohistochemical confirmation. This is particularly important because gastrointestinal symptoms alone are not reliable predictors of gastrointestinal involvement, and the disease may initially grow in the submucosa, making diagnosis challenging when mucosal findings are subtle or nonspecific [4,7]. In the anorectal region, the diagnostic complexity increases because rectal bleeding, anal pain, ulceration, fissures, condylomatous disease, and proctitis can mimic or coexist with malignant disease. In patients with HIV infection, concurrent anal cancer must also be considered, particularly in the presence of persistent bleeding or irregular anorectal mucosa [8]. We present a case of gastrointestinal Kaposi sarcoma with circumferential anorectal involvement and concurrent anal cancer in a young male with controlled HIV infection.

## Case presentation

A 25-year-old male with a history of HIV infection, on treatment with bictegravir and with adequate virologic control documented by undetectable viral load, presented to a private medical consultation with anal pain and rectal bleeding of one month’s duration. Colonoscopy was requested and demonstrated multiple irregular ulcers in the distal 10 cm of the rectum. These lesions were covered with fibrin, showed bleeding, and had elevated hyperemic borders. Biopsy revealed a vascular malignancy secondary to human herpesvirus 8 infection, consistent with Kaposi sarcoma. The patient was referred to Medical Oncology and chemotherapy was initiated.

According to the patient’s medical records, HIV infection had been confirmed by Western Blot on February 7, 2019. Serial laboratory studies demonstrated sustained virologic suppression and preserved immune status during follow-up. On May 22, 2019, HIV viral load was undetectable with a CD4 count of 1017 cells/mm³ (reference range: 500-1500 cells/mm³). On October 20, 2021, viral load remained undetectable with a CD4 count of 1723 cells/mm³ and a CD4 percentage of 49.44%. Additional follow-up studies on August 19, 2022 and July 26, 2023 also demonstrated undetectable viral load, with CD4 counts of 1568 cells/mm³ and 931 cells/mm³, respectively.

The patient subsequently presented to the Coloproctology outpatient clinic because of persistent rectal bleeding, anal condyloma, and anal fissure. He reported zero to two bowel movements per day, corresponding to Bristol stool types 5-6, associated with mucus and blood. Bleeding included bright red and dark blood, clots, and post-defecation dripping. 

On proctologic examination, irregular mucosa was identified approximately 4 cm from the anal verge. Anoscopy demonstrated multiple circumferential inflammatory lesions measuring approximately 0.5 cm, associated with purulent discharge and mild bleeding.

**Figure 1:**
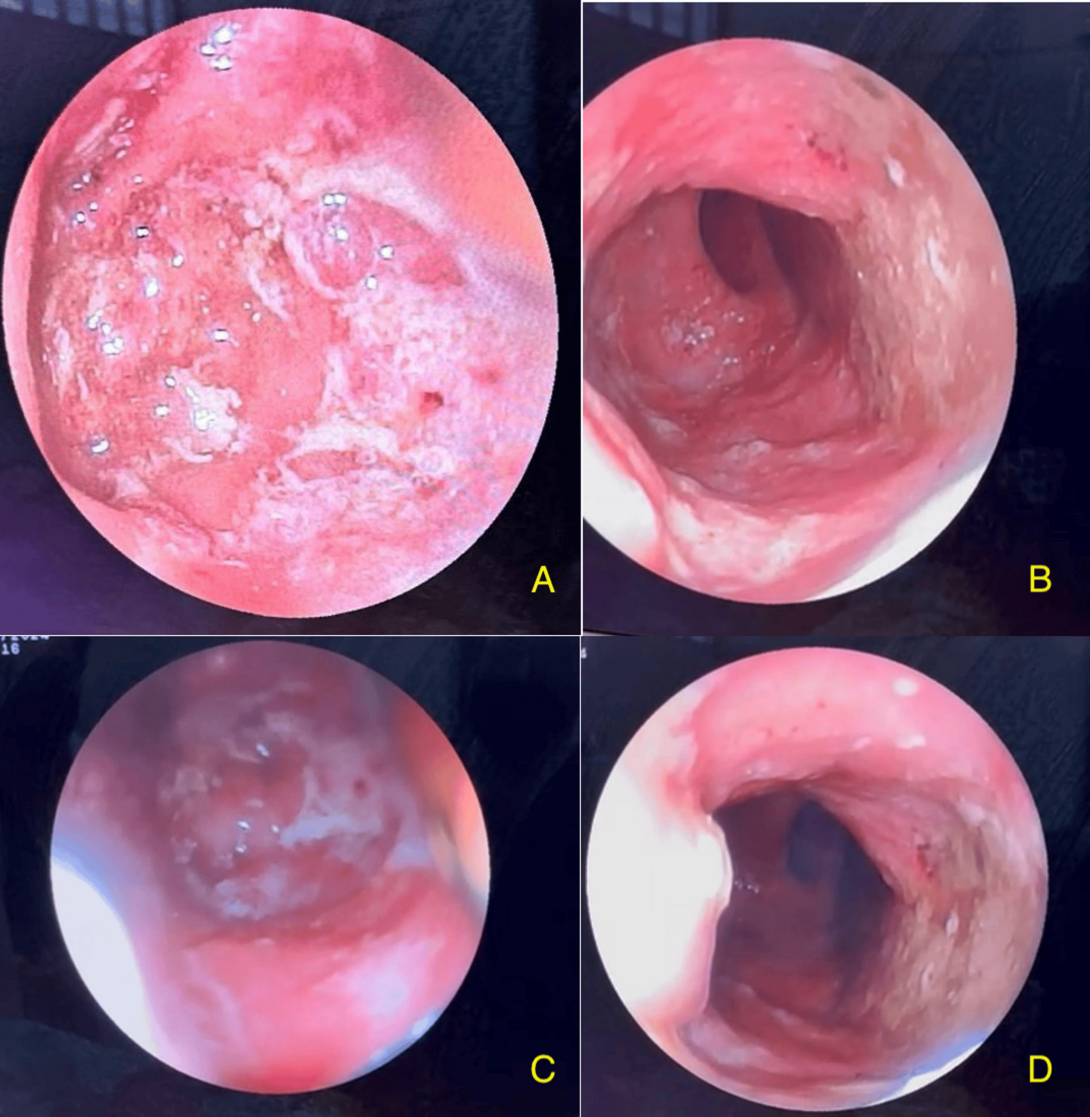
Repeat colonoscopy demonstrated an increased submucosal vascular pattern and elevated, irregular, ulcerated lesions involving the anorectal mucosa circumferentially, extending from the anal canal to 10 cm proximally (A-D).

Given the persistence of symptoms despite initial oncologic treatment, a diagnostic protocol was initiated, including repeat colonoscopy with biopsy in January 2026; irregular areas compatible with tumoral lesions involving the posterior anal margin were identified. Histopathological examination of the anorectal lesions demonstrated spindle-cell vascular proliferation with irregular slit-like vascular spaces and extravasated erythrocytes. Immunohistochemistry was positive for HHV-8, CD31, and CD34, confirming Kaposi sarcoma. Colonoscopy demonstrated adequate rectal distensibility and increased submucosal vascular pattern. The mucosa showed elevated, irregular, ulcerated lesions without active bleeding, extending from the anal canal to 10 cm proximally and involving the entire circumference of the lumen (Figure 1).

A diagnosis of persistent gastrointestinal Kaposi sarcoma persistent to initial medical treatment, with concurrent anal cancer, was established. The patient was referred again to Medical Oncology for multidisciplinary oncologic management. The patient remained under multidisciplinary management with the Infectious Diseases department, which continued antiretroviral therapy with bictegravir/tenofovir alafenamide/emtricitabine, resulting in partial regression of the lesions along with symptomatic improvement. Additionally, the Medical Oncology team initiated adjuvant chemotherapy management.

## Discussion

Kaposi sarcoma is an angioproliferative tumor associated with human herpesvirus 8 infection and is one of the most recognized neoplasms in patients with HIV-related immunosuppression [1]. Gastrointestinal involvement may occur as part of disseminated disease or, less frequently, as the dominant clinical manifestation [2,3]. Although the upper gastrointestinal tract has been more frequently described, lower gastrointestinal and anorectal involvement are well documented and may present with nonspecific symptoms such as diarrhea, abdominal pain, anemia, or overt rectal bleeding [2-4].

The present case is clinically relevant because the dominant manifestation was anorectal disease with persistent anal pain and rectal bleeding in a young patient with HIV infection and undetectable viral load. While gastrointestinal Kaposi sarcoma is classically associated with advanced immunosuppression, the diagnosis should not be excluded solely on the basis of antiretroviral therapy use or virologic control. Lee et al. emphasized that gastrointestinal Kaposi sarcoma may be asymptomatic or present with vague gastrointestinal symptoms, and that endoscopic evaluation is often necessary to establish the diagnosis [2]. Similarly, Tejedor-Tejada et al. described gastrointestinal tract involvement in HIV-associated Kaposi sarcoma and highlighted its endoscopic relevance [3].

The endoscopic pattern in this patient was notable for circumferential elevated, irregular, ulcerated lesions extending from the anal canal to the distal rectum. This finding is important because gastrointestinal Kaposi sarcoma does not have a single characteristic endoscopic appearance. Lesions may appear as reddish or violaceous macules, nodules, polyps, ulcerations, or mass-like lesions with friability and contact bleeding [2,4,7]. Cappell et al. proposed clinical and endoscopic predictors to improve diagnostic recognition of gastrointestinal Kaposi sarcoma, while Nagata et al. showed that gastrointestinal symptoms alone are not sufficient to predict gastrointestinal involvement [4,7]. Therefore, in patients with HIV and persistent anorectal bleeding, endoscopic biopsy should be performed even when symptoms could be explained by benign anorectal conditions.

Several prior reports have described gastrointestinal Kaposi sarcoma as an unusual cause of lower gastrointestinal bleeding. Ling et al. reported recurrent lower gastrointestinal bleeding due to primary colonic Kaposi sarcoma in a patient with AIDS, emphasizing the need to include Kaposi sarcoma in the differential diagnosis of gastrointestinal bleeding in immunocompromised patients [5]. Akanbi et al. similarly described Kaposi sarcoma as an unusual cause of gastrointestinal bleeding, reinforcing that bleeding may be the presenting manifestation rather than an incidental finding [6]. These reports are consistent with the present case, in which persistent rectal bleeding led to repeat proctologic and endoscopic evaluation.

Another important aspect of this case is the anorectal tumoral involvement. In patients with HIV infection, persistent anal pain, rectal bleeding, condylomatous disease, fissures, ulcerated mucosa, or irregular anorectal lesions should not be attributed automatically to benign disease or to known Kaposi sarcoma. HIV-positive patients have an increased risk of anal squamous cell carcinoma, particularly in the setting of HPV-associated disease [8]. Therefore, the presence of anal condyloma and persistent anorectal bleeding in this case made histologic confirmation essential. The coexistence of Kaposi sarcoma and anal cancer also complicates management because each disease has distinct staging, treatment, and prognostic implications.

Garrido et al. described atypical gastrointestinal involvement by Kaposi sarcoma, supporting the concept that this disease may present with extensive or unusual digestive tract findings [9]. The additional literature on atypical gastrointestinal Kaposi sarcoma also includes cases with diffuse gastrointestinal involvement, large rectal masses, isolated rectal lesions, and obstructive complications. These patterns support the need for careful endoscopic inspection and biopsy of suspicious lesions, particularly in immunocompromised patients with persistent or unexplained gastrointestinal symptoms.

Management of HIV-associated Kaposi sarcoma requires optimization of antiretroviral therapy. In symptomatic visceral disease, progressive disease, or cases with significant tumor burden, systemic chemotherapy is frequently considered [10]. However, when anal cancer is present simultaneously, treatment should be individualized through a multidisciplinary approach involving Medical Oncology, Coloproctology, Surgical Oncology, Infectious Disease, and Radiation Oncology. Final treatment decisions depend on the extension of Kaposi sarcoma, response to initial therapy, immune status, histologic subtype and stage of anal cancer, and the patient’s symptoms.

This case has limitations. The available clinical record did not include CD4 count, complete staging workup, imaging findings, chemotherapy regimen, histologic subtype of anal cancer, p16 or HPV status, or longitudinal response to treatment. These data would strengthen the oncologic interpretation and should be added if available before submission. Nevertheless, the case remains valuable because it highlights the importance of repeat endoscopic assessment and biopsy in patients with HIV and persistent anorectal bleeding, even when a prior diagnosis of Kaposi sarcoma has already been established.

## Conclusions

Gastrointestinal Kaposi sarcoma may present with nonspecific anorectal symptoms and mimic benign inflammatory disease, fissure-related bleeding, proctitis, or anorectal neoplastic processes. In patients with HIV infection, persistent rectal bleeding, ulcerated anorectal lesions, or circumferential mucosal involvement should prompt endoscopic evaluation with biopsy and immunohistochemical confirmation. This case highlights the diagnostic challenge of atypical anorectal Kaposi sarcoma even in patients with preserved immune status and undetectable viral load. Multidisciplinary evaluation remains essential for diagnosis, staging, and treatment planning.

## References

[1] Cesarman E, Damania B, Krown SE, Martin J, Bower M, Whitby D (2019). Kaposi sarcoma. Nat Rev Dis Primers.

[2] Lee AJ, Brenner L, Mourad B, Monteiro C, Vega KJ, Munoz JC (2015). Gastrointestinal Kaposi's sarcoma: case report and review of the literature. World J Gastrointest Pharmacol Ther.

[3] Tejedor-Tejada J, Núñez Rodríguez H, Madrigal Rubiales B, González-González D (2020). Kaposi sarcoma involving gastrointestinal tract in VIH. Dig Liver Dis.

[4] Nagata N, Shimbo T, Yazaki H (2012). Predictive clinical factors in the diagnosis of gastrointestinal Kaposi's sarcoma and its endoscopic severity. PLoS One.

[5] Ling J, Coron R, Basak P, Jesmajian S (2013). Recurrent lower gastrointestinal bleeding due to primary colonic Kaposi's sarcoma in a patient with AIDS. Int J STD AIDS.

[6] Akanbi O, Saleem N, Maddika S, Saba R (2016). Kaposi sarcoma: an unusual cause of gastrointestinal bleeding. BMJ Case Rep.

[7] Cappell MS, Nojkov B, Amin M (2015). Proposed clinical and endoscopic predictors for diagnosis of gastrointestinal Kaposi sarcoma. South Med J.

[8] Dandapani SV, Eaton M, Thomas CR Jr, Pagnini PG (2010). HIV- positive anal cancer: an update for the clinician. J Gastrointest Oncol.

[9] Garrido I, Pacheco J, Coelho R, Macedo G (2022). Kaposi's sarcoma with atypical gastrointestinal involvement. Rev Esp Enferm Dig.

[10] Bower M, Dalla Pria A, Coyle C, Andrews E, Tittle V, Dhoot S, Nelson M (2014). Prospective stage-stratified approach to AIDS-related Kaposi's sarcoma. J Clin Oncol.

